# Supplementary figures and images for: Signaling models for dopamine-dependent temporal contiguity in striatal synaptic plasticity
Source: PLoS Comput Biol. 2020 Jul 23;16(7):e1008078. doi: 10.1371/journal.pcbi.1008078 (PMC7402527; doi:10.1371/journal.pcbi.1008078)

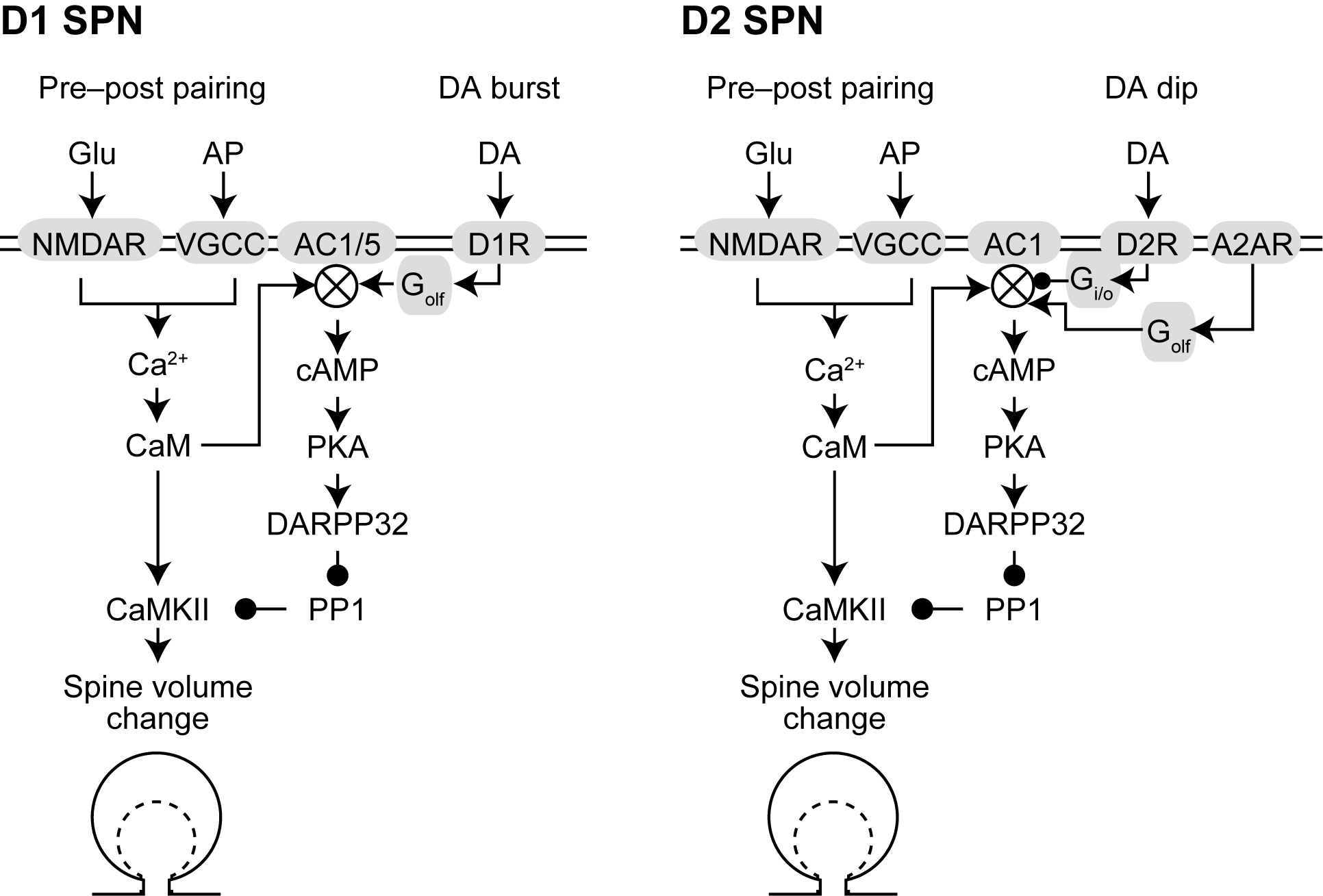

Supplement: S1 Fig — Left: D1 RP model. Pre−post pairing and DA burst/dip were inputs, and PKA activity, CaMKII activity, and synaptic volume change were readouts in the experiment. Right: D2 RP model. Unlike the D1 RP model, DA dip disinhibited AC1 via the relief from Gi/o inhibition, while pre−post pairing and A2AR activated AC1. (TIF) [file pcbi.1008078.s001.tif]

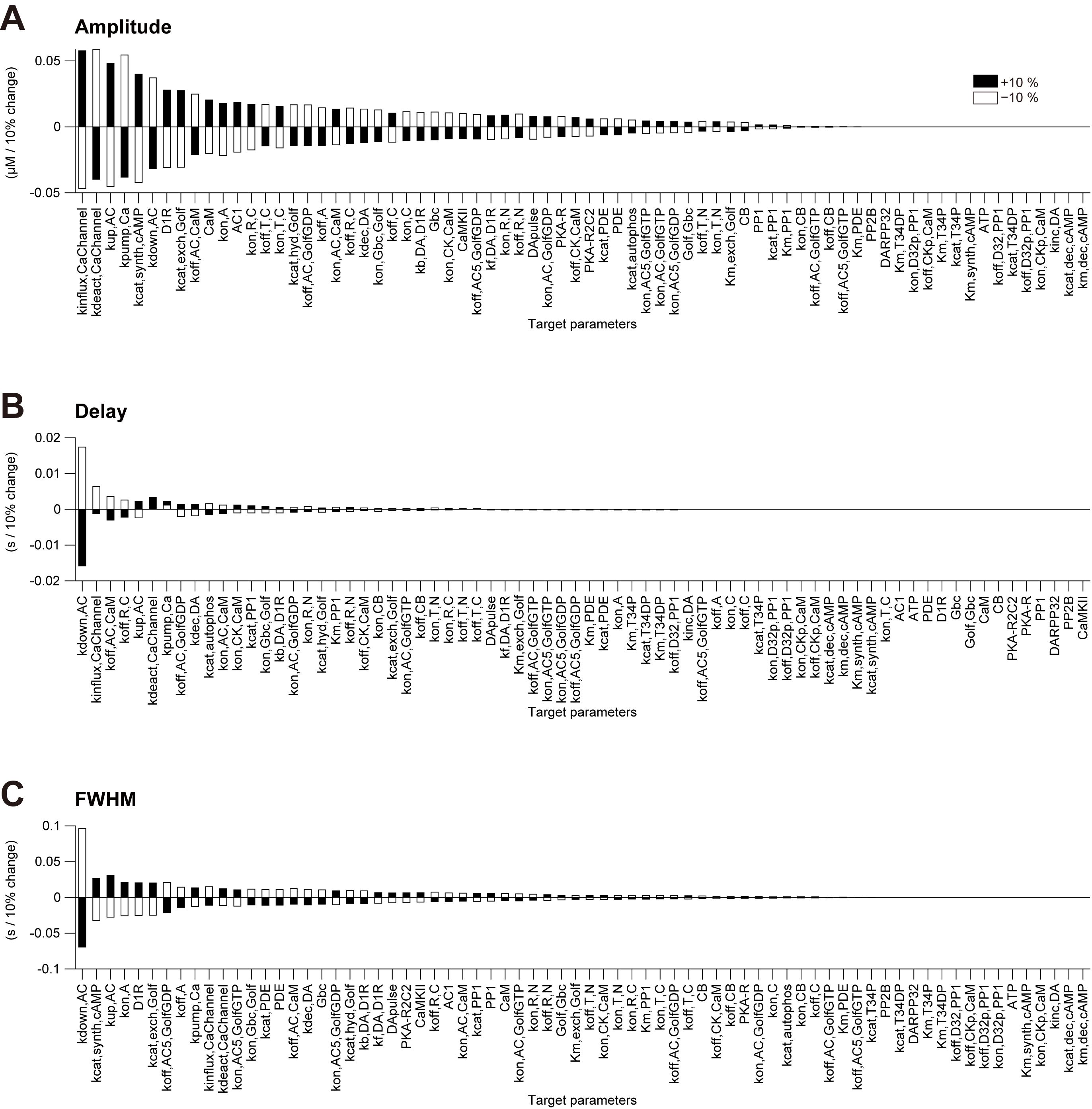

Supplement: S2 Fig — The changes of amplitude (A), delay (B), and FWHM (C) of the time window were quantified when each target kinetics constant or molecular concentration was changed by ±10%. The mean changes of the top twelve changes were plotted in Fig 4. (TIF) [file pcbi.1008078.s002.tif]

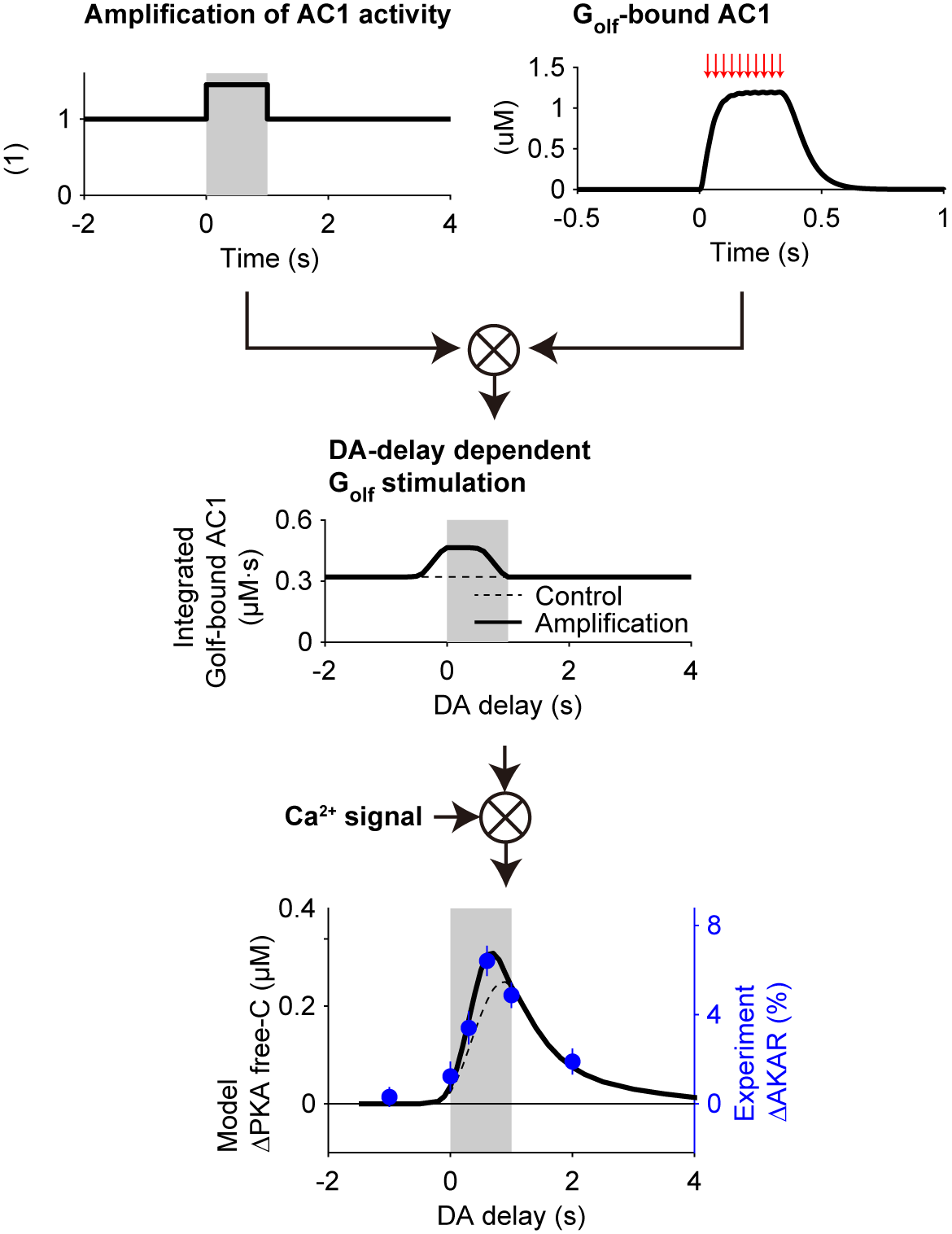

Supplement: S3 Fig — The amplification factor of AC1 activity during pre-post pairing (top, left) was convoluted with DA-dependent Golf stimulation for 0.3 s (top, right). This convolution resulted in the amplification of AC1 between −0.3 s and 0.7 s (middle). The amplification was further multiplied with Ca2+ signal, which resulted in a PKA time window with a 0.6-s peak, as observed in the experiment (bottom, blue). Red arrows denote the times of DA-fiber stimulation (top, right). The amplification level was set to be ×1.45 to give a best fit with the experiment. (TIF) [file pcbi.1008078.s003.tif]
