# Supplementary material for: Signaling models for dopamine-dependent temporal contiguity in striatal synaptic plasticity
Source: PLoS Comput Biol. 2020 Jul 23;16(7):e1008078. doi: 10.1371/journal.pcbi.1008078 (PMC7402527; doi:10.1371/journal.pcbi.1008078)
Supplement: S1 Appendix — Detailed reactions, molecular concentrations, and reaction rate constants. (PDF) [file pcbi.1008078.s004.pdf]

Table A. Molecular concentration

Molecular name	Initial concentration	Notes and references
DA_{basal} (DA_{max})	0.5 μM (10 μM)	Basal DA level is estimated as 0.1-1.0 μM [1, 2]. DA_{basal} was assumed to be 0.5μM. DA-fiber activation gives 10 μM DA within 1 μm from a release site [3-5].
D1R †	0.6 μM	In many cell lines, the molar ratio of receptor:G protein:AC is ~1:100:3 [6]. Also in the striatum, there is 0.3~1 pmol/mg D1R [7, 8], which is two orders of smaller than that of $G_{olf/s}\beta\gamma$ complex (G) [9]. We here set D1R:G = D2R:G = 1:25, because the D1R is specifically expressed at D1R SPNs.
D2R †	0.6 μM	
Adn.A2AR †	0.6 μM	The concentration of A2AR was set to be the same as that of D2R [10]. The extracellular concentration of adenosine (Adn) is 25–250 nM [11], and the affinity of A2AR for Adn is ~10 nM [12]. Thus, A2AR fully binds to Adn.
G † ($G_{olf}\beta\gamma$ complex)	15 μM	The ratio of G_{olf}: $G\beta_2$: $G\gamma_{2,3,7}$ is 72: 220: 530 pmol/mg [7], and the molar ratio of G_{olf}: Gs_{long}: ACs in the striatum is ~27: 16: 1 [13]. Overall, the molar ratio of $G_{olf/s}$: $G\beta\gamma$: ACs is ~27: 80: 1. The amount of $G_{olf/s}$ is similar to that of G_i [14]. No AC5 was assumed in the D2 SPN model, because D2R leads to the continuous activation of AC5, resulting in the large continuous increase in cAMP. We consider that AC5 in D2 SPNs does not function due to an adaptation mechanism.
G † ($G_i\beta\gamma$ complex)	15 μM	
$G_{\beta\gamma}$†	9 μM	
AC1 †	2 μM	
AC5 †	0.14 μM (Spine, D1 SPN), 0.035 μM (Soma, D1 SPN), 0 μM (Spine, D2 SPN)	
RGS †	4 μM	The content of RGS7: RGS9: $G\beta_5$ is 4: 4: 8 fmol/mg in the striatum [15]. Twenty % of total Gβ was assumed to be type 5 [16].
NMDAR †	0 (dimensionless)	Presynaptic spiking leads to the activations of NMDAR and Ca^{2+}-permeable AMPAR [17]. The presynaptic spiking incremented *NMDAR* by one.
VGCC †	0 (dimensionless)	Postsynaptic spiking lead to the activation of VGCC. The consecutive three action potentials incremented *VGCC* by one.
Ca	0 μM	
CaPump †	1.2 μM	Assumption.

CB	120 μM	Faas et al. [18].
N0C0 (CaM)	100 μM	Faas et al. [18].
ATP	2000 μM	Lindgren and Smith [19].
cAMP	0 μM	
PDE †	1.2 μM	The concentration of phosphodiesterase (PDE) was determined to give 30-s PKA activation [20].
R,C	*R*: 5 μM<br>*C*: 8 μM	Neurons have 1 ~ 4 μM PKA [21-23].
DARPP32	50 μM	Bibb et al.[24]
PP1	2 μM	Lindskog et al.[21, 25]
PP2A, *PP2B*	1 μM	
CK	40 μM	~100 μM CaMKII (CK) is expressed in hippocampus [26]. Total CaMKII level in the striatum is ~40% lower than that in hippocampus [27].

† Membrane molecules. The effective cytosolic concentrations of membrane proteins were determined by the multiplication of the indicated concentrations with $SVR = SVR_{\mathrm{target}} / SVR_{\mathrm{spine}}$ where SVR_{target} and SVR_{spine} are the surface-to-volume ratios of the target domain and spine, respectively.

Table B. VGCC/NMDAR-Ca^{2+}- CB dynamics

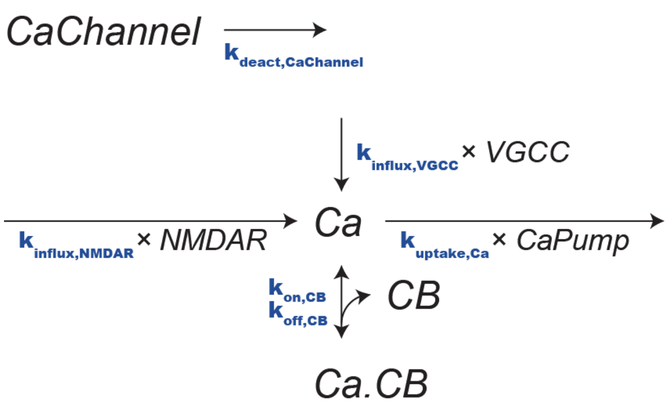

Reaction name	Rate constant	Notes and references
$k_{\mathrm{deact,VGCC}}$	20 s^{-1}	The "CaChannel" represents Ca^{2+} influx via VGCCs/NMDARs. Postsynaptic spiking increased Ca^{2+} level via VGCCs up to ~0.4 μM at SPN spines [17]. Two-photon uncaging of glutamate activates NMDAR and Ca^{2+}-permeable AMPAR, and the Ca^{2+} level increased up to ~2.0 μM [17].
$k_{\mathrm{influx,VGCC}}$	4000 $\mu M^{-1}s^{-1}$	
$k_{\mathrm{uptake,Ca}}$	2200 s^{-1}	Yagishita et al. [20].
$k_{\mathrm{on,CB}}$	75 $\mu M^{-1}s^{-1}$	Faas et al. [18].
$k_{\mathrm{off,CB}}$	29.5 s^{-1}	

Table C. DA-D1/2R-G signaling

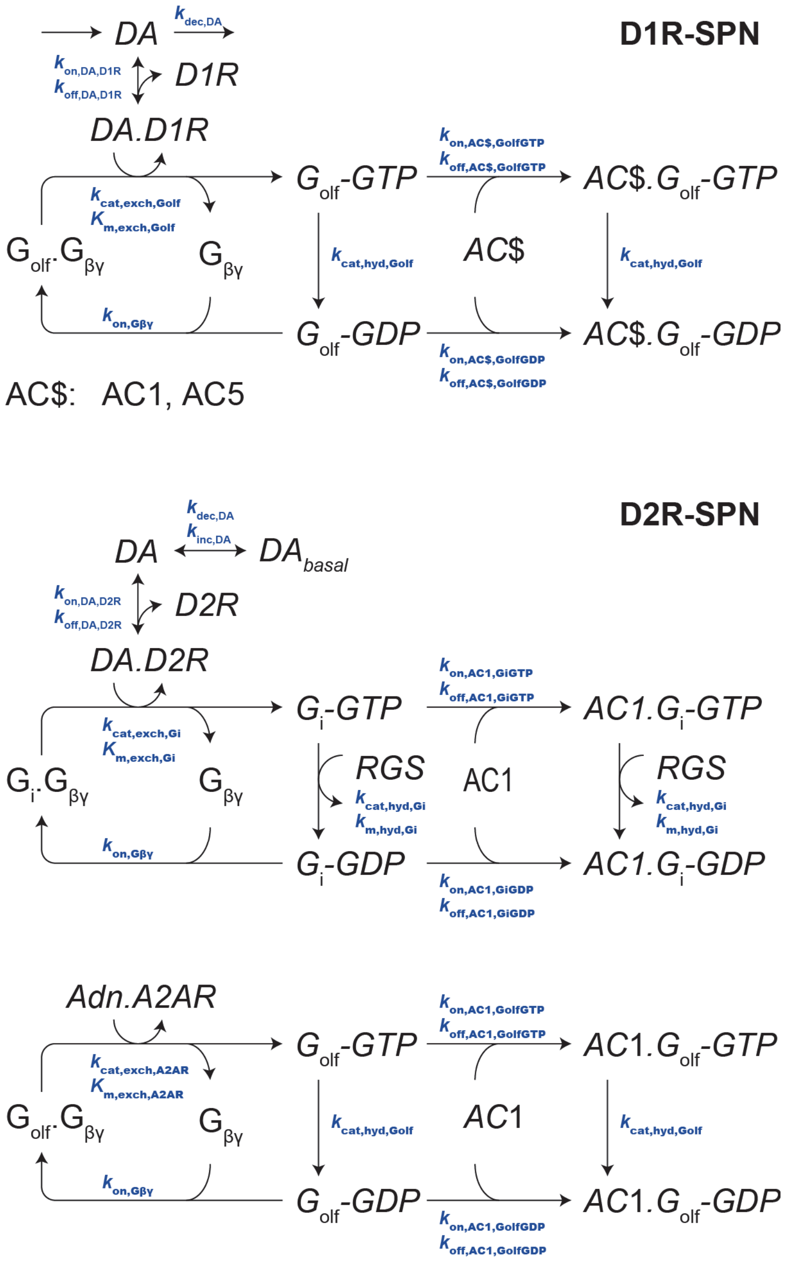

Reaction name	Rate constant	Notes and references
$k_{dec,DA}$	50 s^{-1}	Increased DA level decreases with a time constant of 0.005 s [3-5].
$k_{inc,DA}$	50 s^{-1}	
$k_{on,DA,D1R}$	24 $\mu M^{-1}s^{-1}$	DA binds to D1R and D2R with time constants of $t_{1/2}$ ~ 30 ms [28, 29]. K_ds were determined to satisfy Yapos' experiment [30, 31]. The Kd values (D1R, 2 μM; D2R, 10 μM) were close to those of the low affinity sites of D1R and D2R (4 μM and 2.5 μM, respectively) [32].
$k_{off,DA,D1R}$	50 s^{-1}	
$k_{on,DA,D2R}$	10 $\mu M^{-1}s^{-1}$	
$k_{off,DA,D2R}$	100 s^{-1}	

$k_{cat,exch,Golf}$	67 μM^{-1}s^{-1} /*SVR*	$k_{cat,exch}$ was determined based on [33, 34]. $K_{m,exch}$ is assumed to be saturated.
$K_{m,exch,Golf}$	2 μM **SVR*	
$k_{on,AC1,GolfGTP}$	40 μM^{-1}s^{-1} /*SVR*	G_{olf}-GTP binds to AC1 with a K_d of ~0.1 μM, and G_{olf}-GDP binds with a K_d of ~1 μM [35]. Activation of D1 receptor leads to the activation of G-protein in the range of < 100 ms [31, 36].
$k_{off,AC1,GolfGTP}$	4 s^{-1}	
$k_{on,AC1,GolfGDP}$	40 μM^{-1}s^{-1} /*SVR*	
$k_{off,AC1,GolfGDP}$	40 s^{-1}	
$k_{on,AC5,GolfGTP}$	10 μM^{-1}s^{-1} /*SVR*	Assumption.
$k_{off,AC5,GolfGTP}$	1 s^{-1}	
$k_{on,AC5,GolfGDP}$	10 μM^{-1}s^{-1} /*SVR*	
$k_{off,AC5,GolfGDP}$	10 s^{-1}	
$k_{cat,hyd,Golf}$	50 s^{-1}	$G\alpha_{olf}$ itself shows strong GTPase activity ($t_{1/2}$ < 5 s) [37, 38].
$k_{on,G\beta\gamma}$	10 μM^{-1}s^{-1} /*SVR*	O'Neill et al [39].
$k_{cat,exch,Gi}$	800 μM^{-1}s^{-1} /*SVR*	$K_{m,exch}$ is assumed to be saturated.
$K_{m,exch,Gi}$	0.2 μM^{-1}s^{-1} **SVR*	
$k_{on,AC1,GiGTP}$	40 μM^{-1}s^{-1} /*SVR*	G_i-GTP binds to AC1 with a K_d of ~0.05 μM, and G_i-GDP binds with a K_d of ~2.5 μM [35].
$k_{off,AC1,GiGTP}$	2 s^{-1}	
$k_{on,AC1,GiGDP}$	8 μM^{-1}s^{-1} /*SVR*	
$k_{off,AC1,GiGDP}$	20 s^{-1}	
$k_{cat,hyd,Gi}$	72 s^{-1} /*SVR*	RGS9 shows strong GAP activity for G_t-GTP [40]. The GAP activity of RGS9-2 for G_t are similar to that for G_o [41].
$K_{m,hyd,Gi}$	12 μM **SVR*	
$k_{cat,exch,A2AR}$	80 μM^{-1}s^{-1} /*SVR*	Assumption.
$K_{m,exch,A2AR}$	2 μM **SVR*	

Reactions between membrane molecules were multiplied/divided by *SVR* to give reaction rates per membrane area.

Table D. Ca^{2+}-CaM-AC1 dynamics

Ca^{2+}-CaM binding

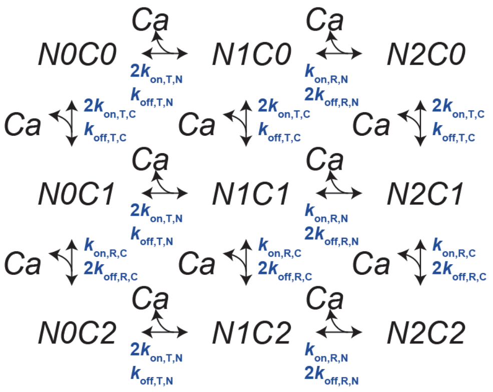

Ca^{2+}-AC1.CaM binding

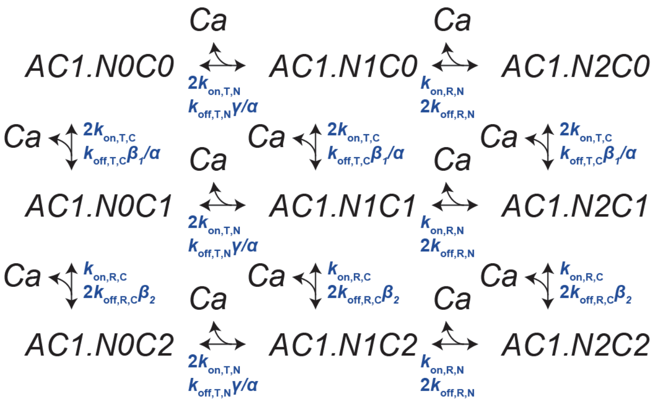

AC1-Ca^{2+}/CaM binding and its state transition

where # ∈ {0, 1, 2} ,
and \$ ∈ {0, 1, 2} ,

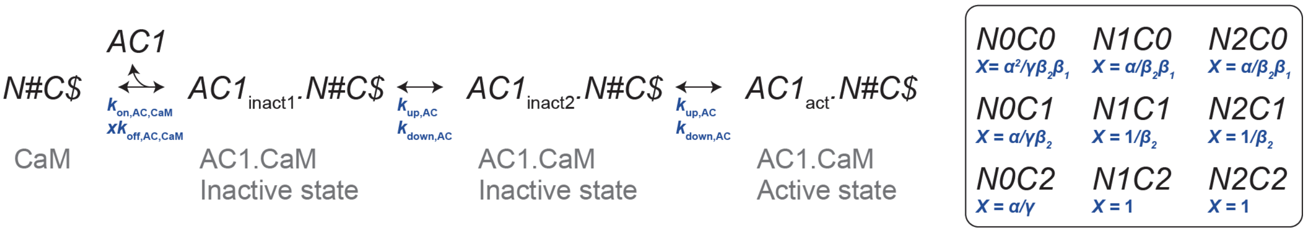

Reaction name	Rate constant	Notes and references
$k_{on,AC,CaM}$	50 $\mu M^{-1} s^{-1}$	The dissociation constant of CaM ($k_{off,AC,CaM}$ / $k_{on,AC,CaM}$) was determined based on Masada et al. (Fig. 3a) [42].
$k_{off,AC,CaM}$	20 s^{-1}	
α	1000	Allosteric regulation factors for CaM binding to Ca^{2+} by AC1. α (= 1000) was introduced to prevent Ca^{2+}-unbound CaM binding to AC1. β_1, β_2, and γ were determined to give $\beta_1 \cdot k_{off,T,C} = 8\ s^{-1}$, $\beta_2 \cdot k_{off,R,C} = 1\ s^{-1}$, and $\gamma \cdot k_{off,T,N} = 8\ s^{-1}$, respectively, for AC1-bound CaM [43].
β_1	3.1×10^{-3}	
β_2	0.104	
γ	5.0×10^{-5}	
$k_{up,AC}$	4.0 s^{-1}	Experiments showed that the activation of AC1 in response to Ca^{2+}/CaM stimulation accompanies a latent time [42, 44, 45]. The latency was modeled as a state transition of CaM-bound AC1.
$k_{down,AC}$	4.0 s^{-1}	
$k_{on,T,N}$	770 $\mu M^{-1} s^{-1}$	Faas et al. [18]. On/off-rate constants for Ca^{2+} binding to CaM N-lobe, T-state.
$k_{off,T,N}$	160000 s^{-1}	
$k_{on,R,N}$	32000 $\mu M^{-1} s^{-1}$	Faas et al. [18]. On/off-rate constants for Ca^{2+} binding to CaM N-lobe, R-state.
$k_{off,R,N}$	22000 s^{-1}	
$k_{on,T,C}$	84 $\mu M^{-1} s^{-1}$	Faas et al. [18]. On/off-rate constants for Ca^{2+} binding to CaM C-lobe, T-state.
$k_{off,T,C}$	2600 s^{-1}	

$k_{\mathrm{on,R,C}}$	25 $\mu M^{-1} s^{-1}$	Faas et al. [18]. On/off-rate constants for Ca^{2+} binding to CaM C-lobe, R-state.
$k_{\mathrm{off,R,C}}$	6.5 s^{-1}	

Table E. AC-cAMP dynamics

$$\begin{cases} f_{AC1} : [\mathit{CaM}\text{-bound } \mathit{AC1}_{act}] = \sum [\mathit{AC1}_{act}.\mathit{N\#C\$}] \text{, where } \# \in \{0, 1, 2\} \text{ and } \$ \in \{0, 1, 2\}, \\ g_{AC1} : [G_{olf/s}\text{-bound } \mathit{AC1}] = [\mathit{AC1}.G_{olf}\text{-}\mathit{GTP}] + [\mathit{AC1}.G_{olf}\text{-}\mathit{GDP}], \\ h_{AC1} : [G_{i}\text{-bound } \mathit{AC1}] = [\mathit{AC1}.G_{i}\text{-}\mathit{GTP}] + [\mathit{AC1}.G_{i}\text{-}\mathit{GDP}], \\ tot_{AC1} : [\mathit{Total\ AC1}] \end{cases}$$

$$\begin{cases} g_{AC5} : [G_{olf/s}\text{-bound } \mathit{AC5}] = [\mathit{AC5}.G_{olf}\text{-}\mathit{GTP}] + [\mathit{AC5}.G_{olf}\text{-}\mathit{GDP}], \\ h_{AC5} : [G_{i}\text{-bound } \mathit{AC5}] = [\mathit{AC5}.G_{i}\text{-}\mathit{GTP}] + [\mathit{AC5}.G_{i}\text{-}\mathit{GDP}], \\ tot_{AC5} : [\mathit{Total\ AC5}] \end{cases}$$

$$[\mathit{Active\ AC}] = \frac{f_{AC1} * g_{AC1} * \{1 - h_{AC1}\}}{tot_{AC1} * tot_{AC1}} + \frac{g_{AC5} * \{1 - h_{AC5}\}}{tot_{AC5}}$$

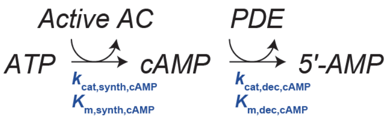

Reaction name	Rate constant	Notes and references
$k_{cat,synth,cAMP}$	100 s^{-1}	Neuronal cAMP is increased up to 1.0~10 μM [46, 47], and we observed PKA response with a PKA-based FRET sensor (AKAR-CR) [20] where AKARs have EC50 values of 0.5~2.0 μM cAMP [48]. Together, $k_{cat,synth,cAMP}$ was set to give ~1.0 μM cAMP. $K_{m,synth,cAMP}$ was set to be much smaller than ATP concentration (~ 2 mM).
$K_{m,synth,cAMP}$	0.1 μM	
$k_{cat,dec,cAMP}$	0.33 s^{-1}	Soderling et al. and Wang et al. [49, 50].
$K_{m,dec,cAMP}$	0.05 μM	

Table F. PKA-DARPP32 dynamics

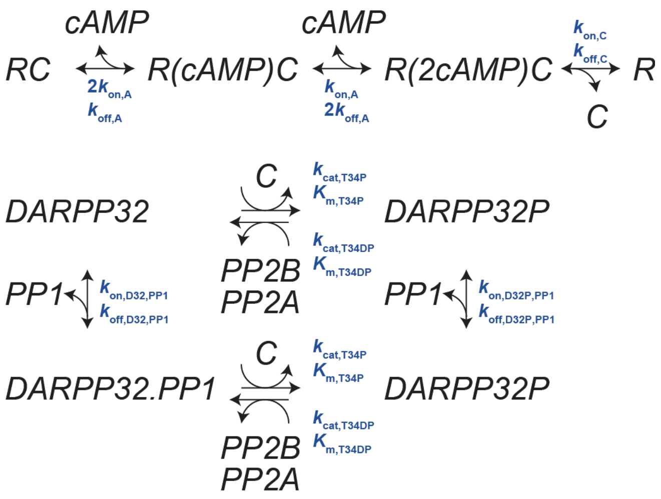

**Reaction name**	**Rate constant**	**Notes and references**
$k_{on,A}$	2 $\mu M^{-1}s^{-1}$	PKA is activated by cAMP with an EC50 of ~2 μM [51], and the Hill coefficient is ~2 [51]. The PKA reporter AKAR (thus PKA) is activated within 5 s in response to cAMP or GPCR stimulations [52, 53].
$k_{off,A}$	10 s^{-1}	
$k_{on,C}$	10 $\mu M^{-1}s^{-1}$	
$k_{off,C}$	40 s^{-1}	
$k_{cat,T34P}$	5.0 s^{-1}	Hemmings et al. for T34 phosphorylation [54], and $K_{m,T34DP}$ = 1.6 μM was taken from King et al. [55]. D1R activity leads to DARPP32 phosphorylation at T34 with a time constant of ~3-10 min in the striatum [56, 57], and DARPP32 is dephosphorylated by PP2B and PP2A [55, 58].
$K_{m,T34P}$	2.4 μM	
$k_{cat,T34DP}$	0.5 s^{-1}	
$K_{m,T34DP}$	1.6 μM	
$k_{on,D32,PP1}$	2 $\mu M^{-1}s^{-1}$	Phorpho-T34 DARPP32 inhibits PP1 with an IC50 value of ~0.7 nM [59, 60], while dephospho-T34 DARPP32 does not inhibit PP1 [60].
$k_{off,D32,PP1}$	0.01 s^{-1}	
$k_{on,D32P,PP1}$	0 $\mu M^{-1}s^{-1}$	
$k_{off,D32P,PP1}$	0.5 s^{-1}	

Table G. PP1-CaMKII dynamics

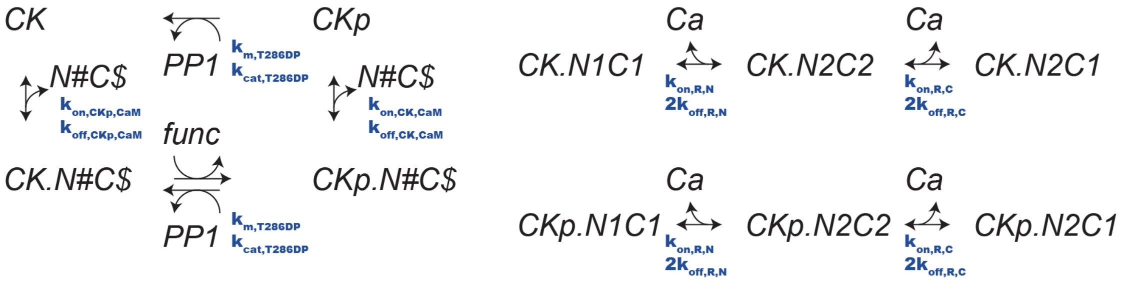

$N\#C\$ \in \{N1C2, N2C1, N2C2\}$

$$[Active\ CK] = (\Sigma\,[CK.N\#C\$] + [CKp.N\#C\$] + [CKp]) / [Total\ CK]$$
$$= ([Total\ CK] - [CK]) / [Total\ CK]$$
$$func = 20 * [Active\ CK]^2 * (-0.220 + 1.826*[Active\ CK] - 0.80*[Active\ CK]^2)$$

Reaction name	Rate constant	Notes and references
$k_{cat,T286DP}$	10 s^{-1}	Bradshow et al. and Urakubo et al. [61, 62]..
$K_{m,T286DP}$	10 μM	
$k_{on,CK.CaM}$	50 $\mu M^{-1}s^{-1}$	Dupont et al. and Meyer et al.[63, 64]
$k_{off,CK.CaM}$	10 s^{-1}	
$k_{on,CKp.CaM}$	50 $\mu M^{-1}s^{-1}$	T286 phosphorylation leads to >1000-fold decrease in the dissociation rate of $Ca^{2+/}$CaM from CaMKII [63, 64].
$k_{off,CKp.CaM}$	0.001 s^{-1}	

Supplementary References

1. Chen KC, Budygin EA. Extracting the basal extracellular dopamine concentrations from the evoked responses: re-analysis of the dopamine kinetics. J Neurosci Methods. 2007; 164(1): 27-42. pmid: 17498808.
2. Lindefors N, Amberg G, Ungerstedt U. Intracerebral microdialysis: I. Experimental studies of diffusion kinetics. J Pharmacol Methods. 1989; 22(3): 141-156. pmid: 2586111.
3. Rice ME, Cragg SJ. Dopamine spillover after quantal release: rethinking dopamine transmission in the nigrostriatal pathway. Brain Res Rev. 2008; 58(2): 303-313. Epub 2008/04/25. pmid: 18433875.
4. Rice ME, Patel JC, Cragg SJ. Dopamine release in the basal ganglia. Neuroscience. 2011; 198: 112-137. Epub 2011/09/24. pmid: 21939738.
5. Arbuthnott GW, Wickens J. Space, time and dopamine. Trends Neurosci. 2007; 30(2): 62-69. Epub 2006/12/19. pmid: 17173981.
6. Ostrom RS, Post SR, Insel PA. Stoichiometry and compartmentation in G protein-coupled receptor signaling: implications for therapeutic interventions involving G(s). J Pharmacol Exp Ther. 2000; 294(2): 407-412. pmid: 10900212.
7. Schwindinger WF, Mihalcik LJ, Giger KE, Betz KS, Stauffer AM, Linden J, et al. Adenosine A_{2A} receptor signaling and G_{olf} assembly show a specific requirement for the γ7 subtype in the striatum. J Biol Chem. 2010; 285(39): 29787-29796. Epub 2010/07/20. pmid: 20639202.
8. Boyson SJ, McGonigle P, Molinoff PB. Quantitative autoradiographic localization of the D1 and D2 subtypes of dopamine receptors in rat brain. J Neurosci. 1986; 6(11): 3177-3188. Epub 1986/11/01. pmid: 3534157.
9. Herve D. Identification of a specific assembly of the g protein Golf as a critical and regulated module of dopamine and adenosine-activated cAMP pathways in the striatum. Front Neuroanat. 2011; 5: 48. Epub 2011/09/03. pmid: 21886607.
10. Schiffmann SN, Fisone G, Moresco R, Cunha RA, Ferre S. Adenosine A2A receptors and basal ganglia physiology. Prog Neurobiol. 2007; 83(5): 277-292. pmid: 17646043.
11. Zhang X, Nagai T, Ahammad RU, Kuroda K, Nakamuta S, Nakano T, et al. Balance between dopamine and adenosine signals regulates the PKA/Rap1 pathway in striatal medium spiny neurons. Neurochem Int. 2019; 122: 8-18. pmid: 30336179.
12. Luthin DR, Olsson RA, Thompson RD, Sawmiller DR, Linden J. Characterization of two affinity states of adenosine A2a receptors with a new radioligand, 2-[2-(4-amino-3-[125I]iodophenyl)ethylamino]adenosine. Mol Pharmacol. 1995; 47(2): 307-313. pmid: 7870039.
13. Zalduegui A, Lopez de Jesus M, Callado LF, Meana JJ, Salles J. Levels of $Gs\alpha_{(short\ and\ long)}$, $G\alpha_{(olf)}$ and $G\beta_{(common)}$ subunits, and calcium-sensitive adenylyl cyclase isoforms (1, 5/6, 8) in post-mortem human brain caudate and cortical membranes: comparison with rat brain membranes and potential stoichiometric relationships. Neurochem Int. 2011; 58(2): 180-189. Epub 2010/12/01. pmid: 21115086.
14. Cai G, Wang HY, Friedman E. Increased dopamine receptor signaling and dopamine receptor-G protein coupling in denervated striatum. J Pharmacol Exp Ther. 2002; 302(3): 1105-1112. pmid:

12183669.

15. Anderson GR, Lujan R, Martemyanov KA. Changes in Striatal Signaling Induce Remodeling of RGS Complexes Containing Gβ5 and R7BP Subunits. Molecular and Cellular Biology. 2009; 29(11): 3033-3044. pmid: ISI:000266006500010.
16. Betty M, Harnish SW, Rhodes KJ, Cockett MI. Distribution of heterotrimeric G-protein beta and gamma subunits in the rat brain. Neuroscience. 1998; 85(2): 475-486. Epub 1998/06/11. pmid: 9622245.
17. Carter AG, Sabatini BL. State-dependent calcium signaling in dendritic spines of striatal medium spiny neurons. Neuron. 2004; 44(3): 483-493. pmid: ISI:000224771700010.
18. Faas GC, Raghavachari S, Lisman JE, Mody I. Calmodulin as a direct detector of Ca^{2+} signals. Nat Neurosci. 2011; 14(3): 301-304. Epub 2011/01/25. pmid: 21258328.
19. Lindgren CA, Smith DO. Increased presynaptic ATP levels coupled to synaptic activity at the crayfish neuromuscular junction. J Neurosci. 1986; 6(9): 2644-2652. pmid: 3018197.
20. Yagishita S, Hayashi-Takagi A, Ellis-Davies GCR, Urakubo H, Ishii S, Kasai H. A critical time window for dopamine actions on the structural plasticity of dendritic spines. Science. 2014; 345(6204): 1616-1620. pmid: ISI:000342164500047.
21. Lindskog M, Kim M, Wikstrom MA, Blackwell KT, Kotaleski JH. Transient calcium and dopamine increase PKA activity and DARPP-32 phosphorylation. PLoS Comput Biol. 2006; 2(9): e119. Epub 2006/09/13. pmid: 16965177.
22. Hofmann F, Bechtel PJ, Krebs EG. Concentrations of cyclic AMP-dependent protein kinase subunits in various tissues. Journal of Biological Chemistry. 1977; 252(4): 1441-1447. Epub 1977/02/25. pmid: 190233.
23. Eigenthaler M, Nolte C, Halbrugge M, Walter U. Concentration and regulation of cyclic nucleotides, cyclic-nucleotide-dependent protein kinases and one of their major substrates in human platelets. Estimating the rate of cAMP-regulated and cGMP-regulated protein phosphorylation in intact cells. Eur J Biochem. 1992; 205(2): 471-481. Epub 1992/04/15. pmid: 1315268.
24. Bibb JA, Snyder GL, Nishi A, Yan Z, Meijer L, Fienberg AA, et al. Phosphorylation of DARPP-32 by Cdk5 modulates dopamine signalling in neurons. Nature. 1999; 402(6762): 669-671. Epub 1999/12/22. pmid: 10604473.
25. Ouimet CC, da Cruz e Silva EF, Greengard P. The alpha and gamma 1 isoforms of protein phosphatase 1 are highly and specifically concentrated in dendritic spines. Proc Natl Acad Sci U S A. 1995; 92(8): 3396-3400. Epub 1995/04/11. pmid: 7724573.
26. Otmakhov N, Lisman J. Measuring CaMKII concentration in dendritic spines. J Neurosci Methods. 2012; 203(1): 106-114. Epub 2011/10/12. pmid: 21985762.
27. Baucum AJ, 2nd, Brown AM, Colbran RJ. Differential association of postsynaptic signaling protein complexes in striatum and hippocampus. J Neurochem. 2013; 124(4): 490-501. Epub 2012/11/24. pmid: 23173822.
28. Lohse MJ, Nikolaev VO, Hein P, Hoffmann C, Vilardaga JP, Bunemann M. Optical techniques to

analyze real-time activation and signaling of G-protein-coupled receptors. Trends Pharmacol Sci. 2008; 29(3): 159-165. Epub 2008/02/12. pmid: 18262662.

29. Patriarchi T, Cho JR, Merten K, Howe MW, Marley A, Xiong WH, et al. Ultrafast neuronal imaging of dopamine dynamics with designed genetically encoded sensors. Science. 2018; 360(6396). pmid: 29853555.

30. Yapo C, Nair AG, Clement L, Castro LR, Hellgren Kotaleski J, Vincent P. Detection of phasic dopamine by D1 and D2 striatal medium spiny neurons. J Physiol. 2017; 595(24): 7451-7475. pmid: 28782235.

31. Marcott PF, Mamaligas AA, Ford CP. Phasic dopamine release drives rapid activation of striatal D2-receptors. Neuron. 2014; 84(1): 164-176. pmid: 25242218.

32. So CH, Varghese G, Curley KJ, Kong MM, Alijaniaram M, Ji X, et al. D1 and D2 dopamine receptors form heterooligomers and cointernalize after selective activation of either receptor. Mol Pharmacol. 2005; 68(3): 568-578. pmid: 15923381.

33. Katanaev VL, Chornomorets M. Kinetic diversity in G-protein-coupled receptor signalling. Biochem J. 2007; 401(2): 485-495. pmid: 16989639.

34. van Unen J, Stumpf AD, Schmid B, Reinhard NR, Hordijk PL, Hoffmann C, et al. A New Generation of FRET Sensors for Robust Measurement of Galphai1, Galphai2 and Galphai3 Activation Kinetics in Single Cells. PLoS One. 2016; 11(1): e0146789. pmid: 26799488.

35. Tang WJ, Stanzel M, Gilman AG. Truncation and alanine-scanning mutants of type I adenylyl cyclase. Biochemistry. 1995; 34(44): 14563-14572. Epub 1995/11/07. pmid: 7578062.

36. Chuhma N, Mingote S, Moore H, Rayport S. Dopamine neurons control striatal cholinergic neurons via regionally heterogeneous dopamine and glutamate signaling. Neuron. 2014; 81(4): 901-912. pmid: 24559678.

37. Brandt DR, Ross EM. GTPase activity of the stimulatory GTP-binding regulatory protein of adenylate-cyclase, Gs - accumulation and turnover of enzyme-nucleotide intermediates. Journal of Biological Chemistry. 1985; 260(1): 266-272. pmid: ISI:A1985TZ26100040.

38. Ross EM, Wilkie TM. GTPase-activating proteins for heterotrimeric G proteins: Regulators of G protein signaling (RGS) and RGS-like proteins. Annual Review of Biochemistry. 2000; 69: 795-827. pmid: ISI:000089735700026.

39. O'Neill PR, Karunarathne WK, Kalyanaraman V, Silvius JR, Gautam N. G-protein signaling leverages subunit-dependent membrane affinity to differentially control betagamma translocation to intracellular membranes. Proc Natl Acad Sci U S A. 2012; 109(51): E3568-3577. Epub 2012/12/06. pmid: 23213235.

40. Martemyanov KA, Arshavsky VY. Kinetic approaches to study the function of RGS9 isoforms. Methods Enzymol. 2004; 390: 196-209. pmid: 15488179.

41. Martemyanov KA, Hopp JA, Arshavsky VY. Specificity of G protein-RGS protein recognition is regulated by affinity adapters. Neuron. 2003; 38(6): 857-862. pmid: 12818172.

42. Masada N, Ciruela A, Macdougall DA, Cooper DM. Distinct mechanisms of regulation by

Ca^{2+}/calmodulin of type 1 and 8 adenylyl cyclases support their different physiological roles. J Biol Chem. 2009; 284(7): 4451-4463. Epub 2008/11/26. pmid: 19029295.

43. Masada N, Schaks S, Jackson SE, Sinz A, Cooper DM. Distinct mechanisms of calmodulin binding and regulation of adenylyl cyclases 1 and 8. Biochemistry. 2012; 51(40): 7917-7929. Epub 2012/09/14. pmid: 22971080.

44. Onyike CU, Lin AH, Abrams TW. Persistence of the interaction of calmodulin with adenylyl cyclase: implications for integration of transient calcium stimuli. J Neurochem. 1998; 71(3): 1298-1306. pmid: 9721756.

45. Yarali A, Nehrkorn J, Tanimoto H, Herz AV. Event timing in associative learning: from biochemical reaction dynamics to behavioural observations. PLoS One. 2012; 7(3): e32885. pmid: 22493657.

46. Mironov SL, Skorova E, Taschenberger G, Hartelt N, Nikolaev VO, Lohse MJ, et al. Imaging cytoplasmic cAMP in mouse brainstem neurons. BMC Neurosci. 2009; 10: 29. Epub 2009/03/31. pmid: 19327133.

47. Muntean BS, Zucca S, MacMullen CM, Dao MT, Johnston C, Iwamoto H, et al. Interrogating the Spatiotemporal Landscape of Neuromodulatory GPCR Signaling by Real-Time Imaging of cAMP in Intact Neurons and Circuits. Cell Rep. 2018; 22(1): 255-268. pmid: 29298426.

48. Sprenger JU, Nikolaev VO. Biophysical Techniques for Detection of cAMP and cGMP in Living Cells. Int J Mol Sci. 2013; 14(4): 8025-8046. Epub 2013/04/16. pmid: 23584022.

49. Soderling SH, Bayuga SJ, Beavo JA. Isolation and characterization of a dual-substrate phosphodiesterase gene family: PDE10A. Proc Natl Acad Sci U S A. 1999; 96(12): 7071-7076. Epub 1999/06/09. pmid: 10359840.

50. Wang H, Liu Y, Hou J, Zheng M, Robinson H, Ke H. Structural insight into substrate specificity of phosphodiesterase 10. Proc Natl Acad Sci U S A. 2007; 104(14): 5782-5787. Epub 2007/03/29. pmid: 17389385.

51. Koschinski A, Zaccolo M. Activation of PKA in cell requires higher concentration of cAMP than in vitro: implications for compartmentalization of cAMP signalling. Sci Rep. 2017; 7(1): 14090. pmid: 29074866.

52. Zhang J, Ma Y, Taylor SS, Tsien RY. Genetically encoded reporters of protein kinase A activity reveal impact of substrate tethering. Proc Natl Acad Sci U S A. 2001; 98(26): 14997-15002. pmid: 11752448.

53. Kocer SS, Wang HY, Malbon CC. "Shaping" of cell signaling via AKAP-tethered PDE4D: Probing with AKAR2-AKAP5 biosensor. J Mol Signal. 2012; 7(1): 4. pmid: 22583680.

54. Hemmings HC, Jr., Nairn AC, Greengard P. DARPP-32, a dopamine- and adenosine 3':5'-monophosphate-regulated neuronal phosphoprotein. II. Comparison of the kinetics of phosphorylation of DARPP-32 and phosphatase inhibitor 1. J Biol Chem. 1984; 259(23): 14491-14497. Epub 1984/12/10. pmid: 6501303.

55. King MM, Huang CY, Chock PB, Nairn AC, Hemmings HC, Jr., Chan KF, et al. Mammalian brain phosphoproteins as substrates for calcineurin. J Biol Chem. 1984; 259(13): 8080-8083. Epub 1984/07/10. pmid: 6330098.

56. Nishi A, Snyder GL, Greengard P. Bidirectional regulation of DARPP-32 phosphorylation by dopamine. J Neurosci. 1997; 17(21): 8147-8155. Epub 1997/10/23. pmid: 9334390.
57. Nishi A, Bibb JA, Snyder GL, Higashi H, Nairn AC, Greengard P. Amplification of dopaminergic signaling by a positive feedback loop. Proc Natl Acad Sci U S A. 2000; 97(23): 12840-12845. Epub 2000/10/26. pmid: 11050161.
58. Nishi A, Snyder GL, Nairn AC, Greengard P. Role of calcineurin and protein phosphatase-2A in the regulation of DARPP-32 dephosphorylation in neostriatal neurons. J Neurochem. 1999; 72(5): 2015-2021. Epub 1999/04/27. pmid: 10217279.
59. Watanabe T, Huang HB, Horiuchi A, da Cruze Silva EF, Hsieh-Wilson L, Allen PB, et al. Protein phosphatase 1 regulation by inhibitors and targeting subunits. Proc Natl Acad Sci U S A. 2001; 98(6): 3080-3085. Epub 2001/03/15. pmid: 11248035.
60. Hemmings HC, Jr., Greengard P, Tung HY, Cohen P. DARPP-32, a dopamine-regulated neuronal phosphoprotein, is a potent inhibitor of protein phosphatase-1. Nature. 1984; 310(5977): 503-505. Epub 1984/08/09. pmid: 6087160.
61. Bradshaw JM, Kubota Y, Meyer T, Schulman H. An ultrasensitive Ca^{2+}/calmodulin-dependent protein kinase II-protein phosphatase 1 switch facilitates specificity in postsynaptic calcium signaling. Proc Natl Acad Sci USA. 2003; 100(18): 10512-10517. pmid: 12928489.
62. Urakubo H, Sato M, Ishii S, Kuroda S. In vitro reconstitution of a CaMKII memory switch by an NMDA receptor-derived peptide. Biophys J. 2014; 106(6): 1414-1420. Epub 2014/03/25. pmid: 24655517.
63. Dupont G, Houart G, De Koninck P. Sensitivity of CaM kinase II to the frequency of Ca^{2+} oscillations: a simple model. Cell Calcium. 2003; 34(6): 485-497. Epub 2003/10/24. pmid: 14572807.
64. Meyer T, Hanson PI, Stryer L, Schulman H. Calmodulin trapping by calcium-calmodulin-dependent protein kinase. Science. 1992; 256(5060): 1199-1202. Epub 1992/05/22. pmid: 1317063.
