# Supplementary material for: Signaling models for dopamine-dependent temporal contiguity in striatal synaptic plasticity
Source: PLoS Comput Biol. 2020 Jul 23;16(7):e1008078. doi: 10.1371/journal.pcbi.1008078 (PMC7402527; doi:10.1371/journal.pcbi.1008078)
Supplement: Attachment — Submitted filename: PCOMPBIOL-D-19-01677_comments.pdf [file pcbi.1008078.s005.pdf]

The manuscript by Urakubo et al et al is trying to shed some light over a very interesting phenomenon related to reward learning and timing requirements. A crucial assumption of the model they propose is that AC1 needs a delay until the Ca/Cam activation kicks in. Thus this window of Ca dependent activation likely decides the optimal window for the G-protein signaling. I was wondering to what extent this can be generalized to timing dependencies in synapses in cortex, hippocampus, etc? Maybe the predicted behavior of a relatively slow Ca/Cam-dependent activation before the onset of Gs/Golf is applicable in other parts of the brain. Is there any data suggesting that it matters if Golf or Gs binds to AC1?

Also please make a sensitivity analysis of the model results to see what aspects of the model that are robust. For instance, can the suggested AC1 timing mechanisms also explain the timing requirements in the publication by Shindou et al (Eur J Neurosci. 2019 49(5):726-736)? If AC1 is activated from the calcium arriving through calcium permeable AMPA receptors, how would that change things? Would the delay in calcium activated AC1 still be possible to keep in similar ranges as in this manuscript?

I assume now that the sensitivity analysis will show that one crucial factor for the model to implement timing sensitivity is indeed that AC1 is assumed to detect Calcium/Cam activity with a certain delay. This is based on the ref by Onyike et al (1998). However, in those experiments 'membrane preparations' were used, and it is difficult to know whether the delay in calcium activation was due to the experimental preparation (e.g. things like diffusion delays in the preparation), and also the calcium signal was strong and quite long-lasting. Probably these uncertainties should be acknowledged, and the suggestion that the timing issues now are completely solved should be toned down. Rather the model predictions could be used as an interesting prediction that if indeed one is searching for a timing sensitivity in AC1 coupled signaling, the AC1 molecule in itself would make the phenomenon to exhibit timing preferences very robust. Perhaps then this can encourage new investigation of the AC1 interactions with calcium/Cam and Golf vs Gs using molecular simulations together with detailed experimental techniques that can work in vivo (i.e. compare the approaches used in Navarro, et al (*Nature Communications* 9, Article number: 1242 (2018),) and Bruce et al.(2019), etc).

Furthermore, when it comes to Yagishita et al., 2014, the comparisons are not necessarily 100% conclusive. Plasticity is shown to be AC5-dependent in striatum (Kheirbeck et al. 2009), and since all corticostriatal and the majority of thalamic synapses are located on dendritic spines AC5 can't be only at somatic or very proximal dendritic regions (as there are no/few spines there). Also the drug used to block AC1, NB001, does not inhibit AC1 directly but has an indirect effect on cAMP accumulation (see Brand et al., 2013), and also inhibits AC5 if concentration is high enough (Wang et al., 2011). To rule out contributions from AC5 one would thus have needed to see that there was no effect on plasticity by inhibiting AC5 more directly. It has also been shown by Lee et al (2002 Journal of Neuroscience 2002, 22 (18) 7931-7940) that AC5 is needed to convey the effects of D2R activation. Indeed it is predicted by Navarro et al (2018) that AC5 is likely already precoupled to the Golf and Gi proteins. Also, it feels in general unlikely that one can disregard significant AC5 contribution in dendrites as up to 80% of the AC activity in striatum is dependent on AC5 (see e.g. Xie et al, eLife 2015, 4:e10451; Kim et al 2014 Mol Brain._7:77). Note that both AC1 and AC5 are membrane bound and the dendritic membranes are large compared to the membrane area

in the soma regions and the most proximal dendrites. These last few points would suggest that even if there are timing effects seen at the level of AC1, this likely needs to be complemented by a timing effect in AC5 signaling as well, otherwise AC5 signaling might shield the timing effects in AC1. This latter point was discussed in Nair et al (2016). Thus the statement that AC1 is dendritic and AC5 somatic should perhaps be reformulated and discussed in a more nuanced way.

Furthermore, one can also point out that the AKAR biosensor (compare Yagishita et al) is not measuring directly PKA, but rather measures the PKA over PPI activities, and PPI can be affected by several calcium dependent factors such as PP2B, PDEs, etc. Thus some of the timing seen in the biosensors can be explained partly by other molecules than PKA.

In summary, I think all these concerns should be discussed openly to encourage future modeling and experimental work to better understand details regarding AC1 and the role of AC5 vs AC1 in the striatum (as well as other synapses).
