# Supplementary material for: Signaling models for dopamine-dependent temporal contiguity in striatal synaptic plasticity
Source: PLoS Comput Biol. 2020 Jul 23;16(7):e1008078. doi: 10.1371/journal.pcbi.1008078 (PMC7402527; doi:10.1371/journal.pcbi.1008078)
Supplement: Attachment — Submitted filename: Response.pdf [file pcbi.1008078.s006.pdf]

Response to the referees' comments

Referee 1:

The manuscript by Urakubo et al is trying to shed some light over a very interesting phenomenon related to reward learning and timing requirements. A crucial assumption of the model they propose is that AC1 needs a delay until the Ca/Cam activation kicks in. Thus this window of Ca dependent activation likely decides the optimal window for the G-protein signaling.

1) I was wondering to what extent this can be generalized to timing dependencies in synapses in cortex, hippocampus, etc? Maybe the predicted behavior of a relatively slow Ca/Cam-dependent activation before the onset of Gs/Golf is applicable in other parts of the brain. Is there any data suggesting that it matters if Golf or Gs binds to AC1?

***Our response*: We appreciate the referee's comment. Yes, timing-dependent RP has been reported in multiple brain regions (He 2015, Neuron 88: 528–538; Shindou 2019, EJN 49:726-736; Brzosko 2015, eLife 4:e09685; Fisher Nat Comm, 8:334, 2017). In particular, two of them seem to share the similar timing-detection mechanism with our RP. Considering the difficulty to generalize the detailed RP models, we newly developed a simple control model that has only two parameters (dead time and first-order delay) (Fig 3B; page 14, lines 300-312), and the simple model was utilized for examining the generalizability of AC1 delay (Fig 9; pages 22-24, lines 523-566). We also discussed the other forms of RP (pages 26-27, lines 615-625), while we did not discuss the difference between Golf and Gs. Here we summarized the points:**

a) **He et al. discovered cortical LTP that has a 5-s time window for norepinephrine (NE) signal (2015, Neuron 88: 528–538; Fig 9C). Because NE activates β_2AR, which is coupled to Gs (similar to Golf), the LTP seems to share similar signaling mechanisms with our RP. Thus, we applied the simple model to the cortical LTP and found the requirement of the longer dead time or first-order delay before AC1-CaM activation (Fig 9C; pages 23-24, lines 559-566).**
b) **Shindou et al. discovered RP in the dorsomedial striatum that has a 2-s time window with a silent period (2019, Eur J Neurosci 49(5):726-736; Fig 9B). The silent period is compatible with the dead time before AC1 activation. We thus applied the simple model to the dorsomedial RP and found the requirement of the longer dead time (Fig 9B; page 23, lines 545-558).**

c) **He et al. also showed cortical LTD that has a 2.5-s time window for serotonin (5HT) signal. However, 5HT activates $5HT_{2C}$Rs, which couples Gq but neither Golf nor Gi. Therefore, the time window should be based on different mechanisms. We stated it (pages 26-27, lines 617-621).**
d) **Brzosko et al (2015, eLife 4:e09685) discovered hippocampal RP that has a 10-30 min time window for DA signal. The very slow dynamics recalls the involvement of completely different signaling, such as cAMP response element binding protein (CREB)-regulated gene expression. We stated it (page 27, lines 621-625).**
e) **Golf are very similar to Gs (Jones and Reed, 1989, Science 244, 790-795), and we do not know their functional difference except the sensitivity against an artificial D1 agonist (DHX; 2018, Nature Comm 9:486). We just touched it (page 26, line 599-601). We are willing to discuss/simulate it if the referee kindly provides information.**

2) Also please make a sensitivity analysis of the model results to see what aspects of the model that are robust. For instance, can the suggested AC1 timing mechanisms also explain the timing requirements in the publication by Shindou et al (Eur J Neurosci. 2019 49(5):726-736)? If AC1 is activated from the calcium arriving through calcium permeable AMPA receptors, how would that change things? Would the delay in calcium activated AC1 still be possible to keep in similar ranges as in this manuscript? I assume now that the sensitivity analysis will show that one crucial factor for the model to implement timing sensitivity is indeed that AC1 is assumed to detect Calcium/Cam activity with a certain delay.

***Our response*: We newly conducted a sensitivity analysis of the D1 RP model, which quantified the effects of each parameter on the peak amplitude, peak time, and FWHM of the time window (Fig 4, pages 15-16, lines 322-351; S2 Fig). The peak amplitude was affected mainly by Ca^{2+}-related parameters, the peak delay was affected by the delay in AC1 activation, and the FWHM was affected by both DA and Ca2+ signals. The sensitivity analysis clarified the importance of delayed AC1 activation, which was modeled as a simple model. We then applied the simple model to Shindous' RP, and found the requirement of the longer dead time of AC1 (Fig 9B; page 23, lines 545-558). Shindous' RP depends on Ca2+ permeable AMPARs. We assumed that the AMPAR-mediated Ca2+ signal similarly contributes to AC1 activation (page 23, lines 546-550).**

3) This is based on the ref by Onyike et al (1998). However, in those experiments ‘membrane preparations’ were used, and it is difficult to know whether the delay in calcium activation was

due to the experimental preparation (e.g. things like diffusion delays in the preparation), and also the calcium signal was strong and quite long-lasting. Probably these uncertainties should be acknowledged, and the suggestion that the timing issues now are completely solved should be toned down.

***Our response*: We appreciate the referee's comment. As the referee pointed out, we just employed the scheme in Onyike et al., but the parameters (dead time, 2.5 s; first order delay, 13 s) were redetermined so as to fit our RP (dead time 0.3 s; first order delay 2 s). The simple model fitting shows that those parameters depend on experiments. We discussed it in the Discussion (pages 25-26, lines 589-602).**

4) Rather the model predictions could be used as an interesting prediction that if indeed one is searching for a timing sensitivity in AC1 coupled signaling, the AC1 molecule in itself would make the phenomenon to exhibit timing preferences very robust. Perhaps then this can encourage new investigation of the AC1 interactions with calcium/Cam and Golf vs Gs using molecular simulations together with detailed experimental techniques that can work in vivo (i.e. compare the approaches used in Navarro, et al (Nature Communications 9, Article number: 1242 (2018),) and Bruce et al.(2019), etc).

***Our response*: We examined brain-region dependence of AC1 activation, and found that the dead time or/and first-order delay differ depending on brain regions (Fig 9; pages 22-24, lines 523-566). This difference may come from the Golf vs Gs, associated molecules, or spatial arrangements of the molecules. We described them (page 26, line 599-602). Again, we do not know any published information about the functional difference between Golf and Gs. We are willing to model/discuss it if the referee kindly provides information.**

5-1) Furthermore, when it comes to Yagishita et al., 2014, the comparisons are not necessarily 100% conclusive. Plasticity is shown to be AC5-dependent in striatum (Kheirbeck et al. 2009), and since all corticostriatal and the majority of thalamic synapses are located on dendritic spines AC5 can't be only at somatic or very proximal dendritic regions (as there are no/few spines there). Also the drug used to block AC1, NB001, does not inhibit AC1 directly but has an indirect effect on cAMP accumulation (see Brand et al., 2013), and also inhibits AC5 if concentration is high enough (Wang et al., 2011). To rule out contributions from AC5 one would thus have needed to see that there was no effect on plasticity by inhibiting AC5 more directly. It has also been shown by Lee et al (2002

Journal of Neuroscience 2002, 22 (18) 7931-7940) that AC5 is needed to convey the effects of D2R activation. Indeed it is predicted by Navarro et al (2018) that AC5 is likely already precoupled to the Golf and Gi proteins. Also, it feels in general unlikely that one can disregard significant AC5 contribution in dendrites as up to 80% of the AC activity in striatum is dependent on AC5 (see e.g. Xie et al, eLife 2015, 4:e10451; Kim et al 2014 Mol Brain. 7:77). Note that both AC1 and AC5 are membrane bound and the dendritic membranes are large compared to the membrane area in the soma regions and the most proximal dendrites. These last few points would suggest that even if there are timing effects seen at the level of AC1, this likely needs to be complemented by a timing effect in AC5 signaling as well, otherwise AC5 signaling might shield the timing effects in AC1. This latter point was discussed in Nair et al (2016). Thus the statement that AC1 is dendritic and AC5 somatic should perhaps be reformulated and discussed in a more nuanced way.

***Our response*: We appreciate the referee's comment. We DO NOT claim that AC1 is localized in dendrites whereas AC5 is in somas. We also do not rule out roles of AC5 in synaptic plasticity. AC5 may also contribute to forming the time window in other forms of RP. In our RP, PKA signal was inhibited by the AC1 inhibitor NB001, thus AC1 should work especially for the coincidence detection of RP (page 27, lines 626-636). Why is AC1, but not AC5, dominantly activated in our RP? We speculate that optogenetic stimulation led to short phasic DA activation (0.3 s), this results in spatiotemporally confined Golf signal that only works for AC1 activation. We described it (page 26, lines 631-636). On the other hand, we did not discuss some of the referee's comments due to the following reasons. We ask the referee's understanding.**

a) **In Yagishita et al., the AC1 inhibitor NB001 successfully inhibited the major component of PKA/cAMP signal. There is no specific reason to describe that NB001 is unreliable because of its indirectness, although any pharmacological agent is not free from side effects.**
b) **Breakdown of inhibitor specificity at high concentration is not a NB001 specific problem, but a common property of pharmacological inhibitors. We carefully determined the concentration of NB001 (50 uM) only for AC1 (Ki: 10 uM) but not for AC5 (Ki: 210 uM).**
c) **Xie et al. discovered the partial coupling and co-expression of $G_{olf}\beta_2\gamma_7$ and AC5 (eLife 2015, 4:e10451), but we do not know its functional consequence.**
d) **As the referee implicitly accepts it, the role of timing detection of AC1 is preserved even if the inhibition of AC5 disrupts RP. AC5 should also play some roles in synaptic plasticity.**

5-2)Furthermore, one can also point out that the AKAR biosensor (compare Yagishita et al) is not

measuring directly PKA, but rather measures the PKA over PPI activities, and PPI can be affected by several calcium dependent factors such as PP2B, PDEs, etc. Thus some of the timing seen in the biosensors can be explained partly by other molecules than PKA.

***Our response*: First, in our experiments, Ca2+ signal did not depend on DA delay (Fig. 3AB and Fig. S6ABC in Yagishita et al., 2014) unlike the Nair's model (2016, PLoS Comput Biol 12: e1005080). Even in this situation, one may think that timing-dependent AKAR activity can be generated by the timing-dependent balance between "DA-triggered activator PKA" and "Ca2+-triggered deactivator PP1". However, AKAR is activated by PKA with a time constant of 5~10 s (Fig. 2C, increase phase), and deactivated by PPX with a time constant of ~15 s (Fig. 2C, decrease phase). They are too long to explain the 2~3-s time window. We thus do not find any reason to cast a doubt on the reliability of the PKA (/PPX) sensor AKAR.**

5-3) In summary, I think all these concerns should be discussed openly to encourage future modeling and experimental work to better understand details regarding AC1 and the role of AC5 vs AC1 in the striatum (as well as other synapses)

***Our response*: We appreciate the referee's comment. As stated above, we discussed possible contribution of AC1 and AC5 in RP (page 27, lines 626-636).**

Referee 2:

1) Overall, what do we learn from the mathematical model in the paper? The presence of AC1 in the dendrites means that both Ca and DA-Golf are needed to trigger PKA-cAMP signaling, so of course, PKA signaling is significant only when the electrical pairing stimulation is superimposed to/coincident with DA stimulation. Since this electrical stimulation lasts for 1 s here (10 pairings @ 10 Hz), the window of efficient DA application is thus expected to be roughly of 1 second after the beginning of the electrical stimulation. In my opinion, this is very much expected and I think there is no need for a mathematical model for that. Since the critical time window of DA application is the major focus of the paper, I am not convinced the new information obtained with the model is enough for publication in PLoS Comput Biol.

***Our response*: We understand the referee's criticism. We did not sufficiently clarify the merits of modeling in the previous manuscript. Encouraged by the editor's suggestion (model prediction), we newly showed "<u>what we can learn from the model</u>" through replying the comments raised by the referee #1. Main components of the revisions are as follows:**

1. **Development of simple models and their application to two other forms of RP.**
   Although the detailed D1/D2 RP models can connect molecules with phenotypes, their complexity makes it difficult to understand the roles of AC1, that is, the first order delay and dead time before AC1 activation. For example, the referee's expectation <u>"Since this electrical stimulation lasts for 1 s here, the window of efficient DA application is thus expected to be roughly of 1 second after the beginning of the electrical stimulation"</u> is not always true. Shindou et al. discovered striatal RP that requires a 2-s DA delay after 0.3-s pre-post pairing, i.e., it accompanies a silent interval (Fig 9B; Eur J Neurosci 49(5):726-736, 2019), and the silent interval can also be explained by the scheme "dead time against Ca2+ stimulation". To clarify this, we newly developed a simple model that has only two parameters (Fig 3B; page 14, lines 300-312), which was applied to our RP as well as two other types of RP (Fig 9; pages 22-24, lines 523-566). The simple model successfully explains all of them, while they require their specific parameters. This predicts that the AC1 coincidence detection depends on brain regions.

2. **Parameter sensitivity analysis.**
   Although AC1 plays central roles, some may think that the other components may play the similar roles. We thus conducted a sensitivity analysis to clarify roles of all parameters in forming the time window (Fig 4, pages 15-16, lines 322-351; S2 Fig).

3. **We proposed a scenario of how the peak can appear at a DA delay of 0.6 s (page 26, lines 603-614; S Fig 3).**

Also, please understand that two preceding models were built to explain the time window for RP (Nakano et al. 2013, Front. Comput. Neurosci. 7:119; Nair et al., 2016, PLoS Comput. Biol. 12(9): e1005080), both of which are based on different molecular mechanisms. So, the idea "AC1 coincidence detection" itself has not been commonly accepted. We described it more explicitly (page 4, lines 73-77). Overall, we believe that the manuscript is now suitable for publication in PLoS Comput Biol.

2) To be fair, I could accept the argument that the interest here is not only on the duration of the critical time window, but also on the exact shape of the dependence of the signaling amplitude to DA delay. Indeed, it is stressed out by the authors that this dependence forms an asymmetric peak where:
   i) there is no PKA signal if both stimulations (electrical and DA) starts simultaneously (zero DA delay).
   ii) PKA signaling remains strong even when the electrical stimulation is finished; in fact, experimentally, PKA signaling seems to remain significant even for 2s delays ie when DA arrives more than 1s after the last electrical pairing.

   While I agree those are puzzling observations, I am not convinced by the interest of the submitted article regarding those points.

2A) First, in terms of experiments, the dependence of PKA signaling to DA delay (ie Fig 1B in the article) around the DA window is based on too few points to be affirmative of its exact shape. At best, the conclusions of the authors rely on only 5 observed DA delays (0, 0.3, 0.6, 1 and 2s). I think one would need a better sampling of the DA delays in the [0-2s] range to ascertain what is the shape of the dependence.

***Our response*: In Yagishita et al. (2014), we repeated sufficient numbers of plasticity/PKA experiments for each DA delay (n > 20 [plasticity] and n > 11 [AKAR] for each 0, 0.3, 0.6, 1 or 2s). Their small SEMs enabled precise comparison with the computational models, and the amount of data was sufficient to draw current conclusion. Unfortunately, the experimental system is currently closed, and we're unable to shortly reactivate it. This is the reason why we submitted the present study not to an experimental journal but to a computational journal. We**

ask the referee's understanding.

2B) To explain the above observations, the authors' model proposes that the whole shape of the critical time window relies on the Ca2+-bound AC1 response (Fig 5A) that, in the model, shows a long latency after the beginning of the electrical stimulation (peaking at the end of the stimulation) and a very long decay to basal values after the electrical stimulation is finished (more than 3 second). In effect, the proposal here is that the "eligibility trace" consists in the Ca2+-bound AC1 response. However, this proposal has already been made based on experimental observation of the time course of AC1 activation after Ca activation. For instance, Onyke et al, J Neurochem 2002 (cited by the authors as ref 35) already noted 17 years ago that the AC1 response was delayed after the onset of the Ca stimulation, peaked only at termination of the calcium signal, and decayed back to basal only after several seconds. The significance of this particular temporal shape in the framework of delayed rewards was even clearly understood and discussed in the paper. The modelling results presented in the submitted article are mostly an illustration of this idea. But I am sorry that I do not see exactly what new significant information is produced by the submitted paper compared to e.g. Onyke et al.

Our response**: Onyike et al. discovered the dead time and long decay (first-order delay) for AC1 activation against Ca2+ stimulation. Yagishita et al. (2014) and the present study showed that those time lags indeed work in the striatum. However, as stated in the 3rd comment of referee #1, in Onyikes' experiments, the decay time and dead time are <u>13 s (not several second) and ~2.5 s</u>, respectively (Fig. 3 in Onyike et al., 2002), which were not consistent with our RP (<u>2 s and 0.3 s</u>, respectively), as newly discussed (pages 25-26, lines 589-602). Because rate constants often depend on experiments, we re-determined these parameters so as to fit the time window. The fit of the simple model further clarified that the dead time and/or first-order delay seem to depend on brain region (Fig 9; pages 22-24, lines 523-566). The referee's comment "<u>this proposal has already been made based on experimental observation</u>" is true. We revisited the Onyikes' discovery, found it works at least in the striatum, and showed its brain region-dependence for the timing window. This is an important model prediction in the context of classical conditioning (page 26, lines 599-602).**

3) Note that actually, the model is not perfectly emulating the experimental timing window. Notwithstanding the critics expressed above related to the number of DA delay experimental points, in the experiments, the maximal delay is of 0.6 sec (i.e. activation at 0.6 s is larger than at 0.3 or 1 s) whereas in the model the peak delay is 1 s (that is why figure 2C uses two DA delays, one for the model and one for the experiments). This discrepancy should be studied in the paper. It might be a sign that all is not perfectly understood in this system.

***Our response*: We appreciate the referee's suggestion. We proposed a scenario about how the peak can appear at a DA delay of 0.6 s with a supplemental figure. Membrane depolarization is known to amplify AC1 activity, which can be a cause of the 0.6-s peak (page 26, lines 603-614; S Fig 3).**

4) Pushing back the details of the mathematical model to the supplementary information is not suitable for the readership of PLoS Comput Biol. The readers will want to know what exactly is in the model before reading the result section.

***Our response* : We appreciate the referee's suggestion. The Methods section was relocated just before the Results section (pages 6-10, lines 93-203). We avoided referring figures in the Methods, because figures must appear where they are firstly referred (regulation of PLoS Comput Biol), and the way of figure presentation will cause confusion. Although we still think that it is impractical that all the contents of S1 Appendix are moved into the Methods section, we described the model of AC1-Ca2+/CaM binding in this section (pages 8-9, lines 156-167), because it is necessary to understand the main results.**

5) Likewise modelling-inclined readers looking at Figure 2D will immediately wonder how come that PKA is activated with a 2s DA delay, for which DA arrives at a point where the calcium trace has reached back its baseline for more than 1s. Of course the answer comes in Figure 5, but I think this is too long a delay between the two figures. I strongly advice to relocate current Fig 5 just after Fig2, so as to explain PKA activation with 2s DA delay right after illustrating it.

***Our response*: We appreciate the reviewer's suggestion. We relocated the previous Fig. 5 into current Figs 3A and 7A.**

Minor Points

6) It is not clear what is plotted in Fig5A middle: is it **active** Ca/CaM-bound-AC1 or total Ca-boud-AC1 (since AC1 has several states here).

***Our response*: The previous Fig 5AB showed active Ca^{2+}/CaM-bound CaMKII. We described them in new figures (current Figs 3A and 7A).**

7) What causes, in the model, the latency of Ca/CaM-bound-AC1? Is it the addition of the two intermediate inactive states for the AC1-Ca/CaM complex or the overall kinetics of Ca binding to CaM (either alone or in complex with AC1). Line 245 says: "The Ca2+/CaM bound to AC1 with a rate constant of ~1 s, and the Ca2+/CaM-bound AC1 was activated after a latent time of ~0.3 s". That seems to imply that the major part of the latency is due to Ca/CaM binding to AC1 and not to Ca2+/CaM-bound AC1 activation.

***Our response*: We appreciate the reviewer's comment. The deadtime of AC1 was generated by "two intermediate inactive states for the AC1-Ca/CaM complex," and the first order delay of AC1 was generated by both "two intermediate inactive states for the AC1-Ca/CaM complex" and "the overall kinetics of Ca binding to CaM". We revised correspondent description with the simple model (Fig. 3A; page 13, lines 268-284).**

8) Yagishita et al, Science 2014 is not the only experimental paper that questioned the critical time window of DA application after STDP in SPNs. Other papers exist, including Fisher et al, Nature Comm, 8:334, 2017 or Brzosko et al, eLife, 4:e09685, 2015 and must be cited. And their results must be discussed in the light of Yagishita et al, Science 2014 and the results of the present submission.

***Our response*: We appreciate the reviewer's suggestion. We cited them (page 25, line 577; pages 26-27, lines 615-625). In the paragraph, we discussed the relationship of our RP with those forms of RP (pages 26-27, lines 615-625).**

9) I do not understand the bottom panel of Figure 5D: the peak level of PKA Free-C is much lower with 0.6 sec electrical pairing stimulations than with 1 sec ones, even for huge delays, e.g. -6 or + 6 seconds. With such large delays there is no interaction at all between the electrical and the DA stimulations. So there's no reason a 0.6 sec pairings would give such a lower peak of PKA free C

with eg +6 or -6 sec DA delays. If it the difference is real and not an error of the plot, it must be explained.

***Our response*: The basal PKA activation came from the interaction of continuous A2AR and phasic Ca2+ signals upon incompletely Gi-inhibited AC1. The PKA activation was DA-delay independent. We stated it (Fig. 7C; page 21, lines 479-487). The incomplete inhibition produced the decreasable basal activity of PKA, which has been observed in other studies (e.g., Bateup, Nat Neurosci 11(8): 9392-939, 2008), while it caused the instability. They should be reconciled with unknown adaptation mechanisms. We have discussed it (page 30, lines 694-709).**

10) lines 468-469: from these equations, it seems that each prepost pairing increases the VGCC rate and the NMDAR rate by exactly 1 unit. I do not understand how the model can work without a parameter to modulate the amplitude of these increments (as is the case for DA rate for instance).

***Our response*: We newly made a parameter sensitivity analysis (Fig 4, pages 15-16, lines 322-351; S2 Fig), which shows the effects of all parameters on the time window. The NMDA/VGCC was increased by exactly 1 unit. The change of this amplitude is mathematically equivalent to the change in the rate of Ca2+ influx, $k_{\mathrm{influx,\ CaChannel}}$. We briefly stated it (page 16, lines 341-342).**

We noticed that, in the submitted version, NMDAR/VGCC-mediated Ca2+ influxes were collectively modeled as a single variable "CaChannel." We corrected it, and apologize for the confusion. Experimentally observed Ca2+ signal was fairly reproduced in this Ca2+ model as shown below. We are willing to show the figure panels if the referee thinks that it is important for validity.

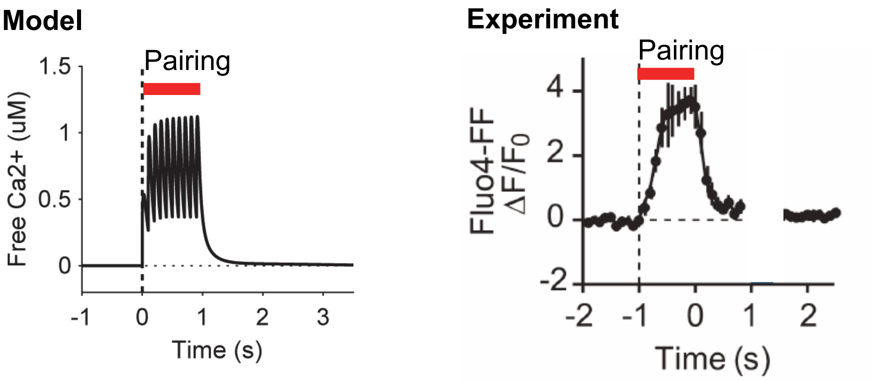

11) Fig 6C: around line 290, the authors claim that the FWHMs of the duration of the CaMKII response is larger than that of the PKA response in Fig 6C. This would agree with their model predictions. Unfortunately, in the absence of any quantification of figure 6C, I must say that this claim is not at all obvious from visual inspection of the figure.

***Our response*: We appreciate the referee's comments. Because FWHMs could not be measured in the experimental data, we fitted exponential decay functions to the AKAR and Camui activities, and the significantly longer activity of CaMKII was confirmed (Fig 8C, D; page 21, lines 495-499; page 10, lines 185-189).**

12) The manuscript indicates that the Matlab code used to simulate the model is available on a github repository. Unfortunately, the corresponding url does not seem valid, so that it is not possible to check the simulation code (I have found the code for a previous paper on the github of the first author, but not for the submitted paper).

***Our response*: The Matlab code and SBML files were uploaded to the Github site (https://github.com/urakubo/ModelRP). We apologize for the inconvenience.**
