# Supplementary material for: Signaling models for dopamine-dependent temporal contiguity in striatal synaptic plasticity
Source: PLoS Comput Biol. 2020 Jul 23;16(7):e1008078. doi: 10.1371/journal.pcbi.1008078 (PMC7402527; doi:10.1371/journal.pcbi.1008078)
Supplement: Attachment — Submitted filename: ResponseToReviewers.pdf [file pcbi.1008078.s007.pdf]

Response to the reviewers' comments

Reviewer #1:

The authors have tried to address most of the previous concerns, so I'm happy to approve the publication. This is an important paper as it addresses a very important phenomenon that is important for reward learning.

***Our response*: We appreciate the reviewer's comment. We are pleased to hear your approval of publication.**

Reviewer #2:

MAJOR POINTS

1) The authors now systematically refer to AC1 stimulation as having two types of time lags (dead time and first-order delay). I understand this necessity because of the equation for the "simple model" shows two constants ¥tau and T. But for the reader who is not an expert in dynamics systems, it must be explained what these two-time lags correspond to, in terms of dynamics. In particular, it must be explained that the "first-order delay" also sets the time-scale of the decay of the AC1 signal once the calcium signal is off. Moreover, I believe that the usual term in dynamical systems textbooks is not "first-order delay" but "first-order time constant".

***Our response*: We appreciate the reviewer's suggestion. We added a description of the roles of the "first-order time constant" in AC1 signal (page 13, lines 261-265). Also, the first-order time constant was shown in the revised Fig 3A (left, red dotted lines). The term "first-order delay" was replaced by "first-order time constant."**

2) Figure 2C: The model for "DA+pairing" shows a dead-time that is similar to the experimental curve. However, the experimental data for "DA only" also exhibits a similar dead-time, a feature that is not reproduced by the model (no dead time in the model for "DA only"). I believe this might be a hint that the hypothesis made by the authors that the dead time originates entirely from AC1 activation by Ca2+/CaM may be flawed. Indeed, if the dead time survives in the absence of Ca2+/CaM stimulation, one may reasonably argue be that the dead-time is not due to Ca2+/CaM activation of AC1. I believe that the current manuscript does not treat this point seriously enough.

***Our response*: If the dead time in DA signal, T_{DA}, is introduced, the dead time of Ca^{2+}, T_{Ca}, should be increased to $T_{Ca} + T_{DA}$ to obtain the same change in synaptic strength, ΔSynaptic strength(t_{delay}). Simply,**

$$\Delta\text{Synaptic strength}(t_{\text{delay}}) = \int_{-\infty}^{\infty} AC(t, t_{\text{delay}})\, dt$$

$$= \int_{-\infty}^{\infty} \text{Processed\_Ca}(t - T_{Ca}) \cdot DA(t, t_{\text{delay}})\, dt$$

$$= \int_{-\infty}^{\infty} \text{Processed\_Ca}(t' - \{T_{Ca} + T_{DA}\}) \cdot DA(t' - T_{DA}, t_{\text{delay}})\, dt',$$

where $t = t' - T_{DA}$. Thus, the introduction of DA dead-time does never violate the requirement of Ca^{2+} dead time. We stated this (page 15, lines 311-316). Any PKA (thus AC) increase was not observed in the absence of Ca^{2+}/CaM stimulation (Fig 2C).

3) Simple model for AC1 response:

3-a) I understand that the equation given line 305 is written for simplicity in terms of its Laplace transform, but I think it would be very useful for the less signal processing-inclined readers to also give the inverse transform of the first term of the RHS member for e.g. a square-pulse calcium, i.e. $¥theta(t-T) (1-¥exp(-(t-T)/¥tau)) - ¥theta(t-T-d) (1-¥exp(-(t-T-d)/¥tau))$ where d is the duration of the square-pulse calcium input and $¥theta$ is the Heaviside function.

***Our response*: We appreciate the reviewer's comment. We described the inverse transformed form (page 14, lines 300-304).**

3-b) the conventions used in Figure 9 are at odds with the other figures of the paper. That is quite misleading. For instance, in Figure 1-8, the delay between pre-post stimulation/Ca signal and DA application is counted starting from the beginning of the pre-post stimulation/Ca signal whereas the "interval" of figure 9 starts at the end of the pre-post stimulation/Ca signal. As a result, for the NAc data, the plasticity window is [0,4] seconds in Fig 1-8 but [-1,3] seconds in Figure 9. Moreover, the Ca-DA delay is most of the times referred to as "DA delay" (Fig 1,2,4,5,8), but sometimes "¥Delta t" (Fig 3B, 7B), "DA-burst delay" (Fig 3C) or "Interval" (Fig 9). Please homogenize notations.

***Our response*: The time-axes were re-written as "DA delay" in Fig 3B, C, and "DA-dip delay" in Figs 6, 7, and 8E, right. As stated below (3-c, d), we removed Fig 9 and relevant sentences.**

3-c) There seems to be potential inconsistencies between the bottom line of figure 9 and the main text. In B, the figure reads '$¥tau=2 s, T=2.14 s$ whereas the main text indicates that the best fits were obtained with $T=5$ and (probably independent fit) $¥tau = 0.6s$. Likewise, for C, the figure says $¥tau=2 s$ whereas the main text instead points to $¥tau = 5.06$. Moreover, I think it would help the reader if Fig 9 would also plot the delayed AC1 signal (i.e. the inverse Laplace transform of the equation line 305), in addition to the Ca and DA pulses/signals of the top line. That would be useful to realize in practice what the estimated parameter values actually mean.

3-d) While I acknowledge the value of inclusion of the "simplified model", I believe the authors should be more cautious with their interpretation of the fits of Fig 9. First, the number of data points used for the fits is really very very small (4 points for the DMS, only 3 points for the cortex!). Moreover, the values of the estimated parameters, especially the dead-time, are very large (from 2 to 7 s). It is difficult to understand how the simple signaling mechanism of Fig 1C could give rise to such large dead-times. Note that I am not saying that this figure should be removed from the paper, but that the conclusions drawn from it should be taken with a (large) pinch of salt.

***Our response*: We plotted delayed AC1 signals according to the reviewer's suggestion (solid lines in the top panels of Fig 9; pages 5-7 of this letter). As the reviewer pointed out, the large dead times certainly produced a bit unnatural AC1 signals, which were strongly affected by additional datapoints. Considering the benefit and disadvantage of this simple model fitting, we decided to remove Fig 9 and relevant description completely from the manuscript (pages 5-7 of this letter). The abstract, summary, introduction, and discussion were also revised (page 2, line 39; page 3, line 52; page 5, line 90; page 24, lines 558 and 562). This was an extended analysis, and the conclusion of the present study is preserved.**

4) The question of whether AC1 is present (in addition to AC5) in MSNs is central in the paper, and goes against the dogma in the field. In the current manuscript the question is tackled late in the results section (l 242) and in the discussion section, but I think it is important enough to be given in the introduction and not relegated to later sections.

***Our response*: We appreciate the reviewer's comment. We gave a description of the AC1/5 problem in the introduction section (page 4, lines 73-74; pages 5-6, lines 79-81). AC1 is certainly present (expressed) in the striatum (eg., Visel 2006, J Comp Neurol 496:684–697; Zalduegui 2011, Neurochem Int 58, 180–189). We acknowledged it (page 5, line 81).**

MINOR POINTS

- line 30: "two types of time lags". I don't understand the point of specifying here in the abstract that *two* time lags have been considered. If you don't explain there what are those two time lags, this detail is as best confusing.

***Our response*: We simplified it (page 2, lines 32). We appreciate the reviewer's comment.**

- l49: "negative PRE" should be "negative RPE"

***Our response*: We corrected it (page 3, line 50).**

- l71: "D1R/dopamine D2 receptors (D2R)" is cumbersome. Maybe "dopamine D1 (D1R)/ D2 (D2R) receptors" would be clearer

***Our response*: We corrected the phrase "D1R/dopamine D2 receptors (D2R)" into "D1Rs or dopamine D2 receptors (D2Rs)" (page 4, lines 70-71).**

- l96: Fig 1C is referred to here before Fig 1A and B. Please consider correcting this.

***Our response*: We re-ordered the panels in Fig 1 (previous panel C was re-located as panel A). We appreciate**

the comment.

- l123: "All higher order binding reactions (> 3) were decomposed into sets of second-order reactions. This is important because the approximation of higher order binding reactions is inappropriate to simulate temporal dynamics of molecules. Enzymatic reactions were also modeled based on the Michaelis-Menten formulation:". Actually, the Michaelis-Menten formalism is also an approximation to aggregate 3 elementary reactions and is also not very good to simulate temporal dynamics. Ideally, enzymatic reactions should also be decomposed into elementary (first- or second-order) reactions.

***Our response*: As the reviewer pointed out, the Michaelis-Menten formulation is an approximation. The original three elemental reactions (elemental formulation) utilize an enzyme-substrate complex to give a precise production rate. Here, such an enzyme commonly forms complexes with divergent substrates, depending on their concentrations and K_m values, whereas the RP models are only a subset of overall spine signaling. We were thus not convinced the actual dynamics of enzyme-substrate complexes. Also, the elemental formulation introduces an unobservable variable (kf, kb, and kcat estimation only from Km and kcat) and causes computational instability. Altogether, we decided to ignore the factor of enzyme-substrate complexes completely. On the other hand, the introduction of higher-order binding reactions (> 3) often results in a catastrophic slowdown of molecular binding/unbinding dynamics. This can be seen in the binding of 4 Ca2+ ions with CaM, which must be avoided.**

Although the determination of the level of abstraction/approximation is always a problem of a modeling study, we believe that the current RP model is one of acceptable ones. We do not write such details in the manuscript. We would ask the reviewer's understanding.

- l148: "D1Rs were located in the cytosolic domain": I don't see the interest of this information here? Do you refer to the experiments or the model? Since you have a single-compartment, perfectly-mixed model, it's impossible to account for different localizations anyway, so this sentence is at best confusing.

***Our response*: This sentence was removed (page 8, line 162).**

- l201: "ΔWlexp,i" should be "ΔWexp,i"

***Our response*: We removed the correspondent sentence (page 6, line 4 of this letter).**

Removed parts from the previous manuscript

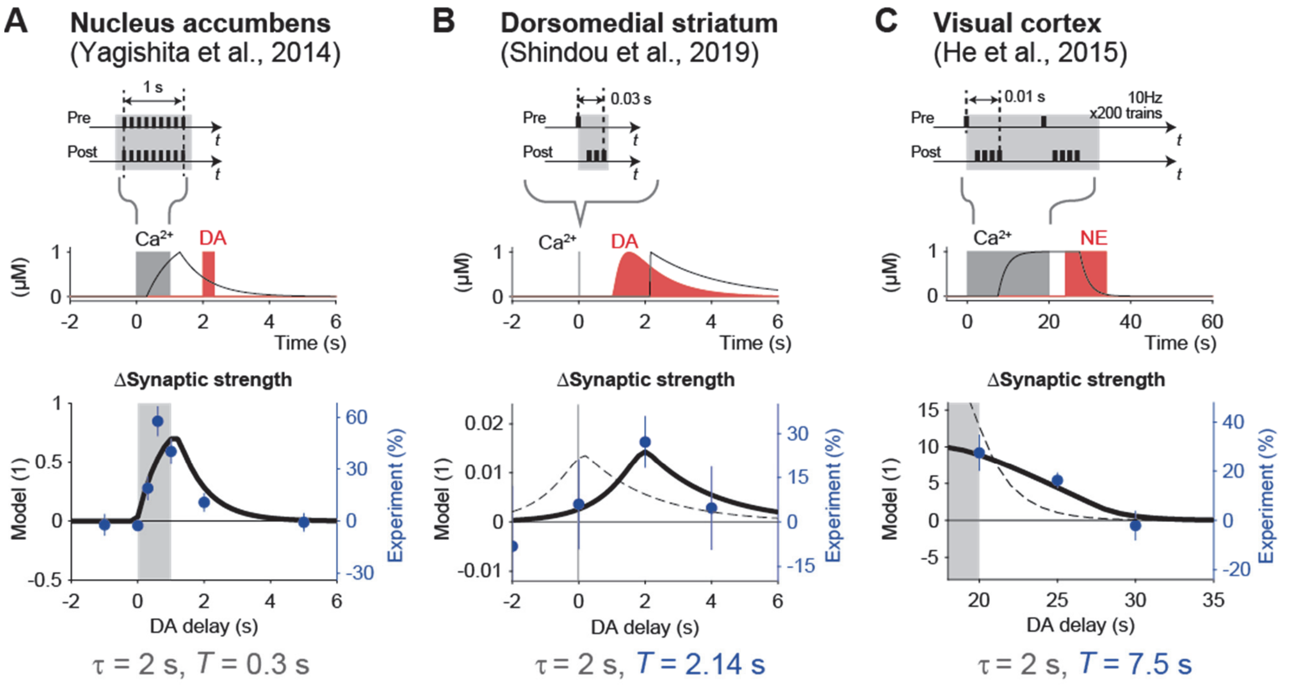

Fig 9. Fitting of the simple model to RP in multiple brain regions.

(A) Simple model and D1 RP in the NAc (bottom) [1]. One-second pre–post pairing and a 0.3-s DA burst were represented by their rectangular elevations (top). Solid black line denotes the profile of active Ca^{2+}/CaM-bound AC1. (B) Simple model fitting to RP in the dorsomedial striatum (thick line, bottom) [2]. The model showed a best fit if T was increased to 2.14 s (bottom). The simple model with the original parameters was also shown (dashed line, $\tau = 2$ s and $T = 0.3$ s). Pre–post pairing was represented by rectangular Ca^{2+} signal (duration: 0.03 s, top), and DA signal was represented by a double exponential function ($\tau_{activation} = 0.25$ s and $\tau_{deactivation} = 1.0$ s, top) where the peak amplitude was normalized to 1. Experimental data were taken from Fig 4D, G of Shindou et al. [2]. (C) Simple model fitting to RP in the visual cortex (thick line, bottom) [3]. The model gave a best fit when T was increased to 7.5 s (bottom). Simple model with the original parameters was also shown (dashed line, $\tau = 2$ s and $T = 0.3$ s). He et al. gave 20-s pairing together with a 10-s norepinephrine (NE) puff. They were represented by the rectangular elevations of Ca^{2+} and NE ($DA(t)$) (top). Experimental data were taken from Fig 6A of He et al. [3].

Methods

Fitting a simple model to RP in the dorsomedial stratum and visual cortex.

We developed a simple D1 model that had only two parameters (see Results), and fitted the model with experimentally observed RP in the dorsolateral striatum and visual cortex [2, 3]. First, the original set of parameters was defined as $\tau = 2$ s and $T = 0.3$ s for RP in the NAc [1]. Then, we conducted least-square fitting to give a best fit with RP in the dorsolateral striatum and visual cortex. Either of the parameters was set to be a subject of the least-square fitting, while the other was fixed at the original one, because the numbers of experimental data points were

too small to perform the fitting of both the parameters. The coefficient of determination (R^2) was utilized as a measure of goodness-of-fit, which is defined by:

$$R^2 = 1 - \frac{\sum_i (\Delta W_{model,i} - \Delta W_{exp,i})}{\sum_i (\Delta W_{model,i} - \overline{\Delta W_{exp,\iota}})},$$

Where $\Delta W_{model,i}$ and $\Delta W_{exp,i}$ are i^{th} data point in the model and experiment, respectively. R^2 becomes close to 1 if the model shows a better fit to experimental data. $\Delta W_{x,i}$ was normalized by the mean amplitude, $\overline{\Delta W_{x,\iota}}$.

Results

Simple model fitting to RP in multiple brain regions

In addition to the NAc, RP has been reported in multiple brain regions [2-5], and at least two of them seem to share the same signaling mechanisms with D1 RP in the NAc (see Discussion) [2, 3]. Because molecular expression depends on brain regions, it is difficult to examine the versatility of AC1 signaling by using the detailed RP model. We thus examined the similarity of the other two forms of RP by using the simple D1 RP model for the NAc (Fig 9A).

We first applied the simple model to D1 RP in the dorsomedial striatum [2]. Shindou et al. reported a form of D1 RP that has a 2-s time window (Fig 9B). Given that both are part of the striatum, the dorsomedial striatal RP should share the similar mechanisms with D1 RP in the NAc, although the dorsomedial striatal RP depends neither on NMDARs nor on L-type VGCCs, but on Ca^{2+}-permeable AMPARs [2]. We assumed the AMAPR-mediated Ca^{2+} signal, which was modeled as a rectangular Ca^{2+} wave, similarly affects AC1. DA signal was modeled by a double exponential function (Fig 9B, top) as observed in their experiment [2]. Upon those inputs, the simple model with original parameters ($\tau = 2$ s, $T = 0.3$ s) showed a time window, but which did not match the time window reported by Shindou et al. (Fig 9B, bottom; dashed line and blue points; R^2, -1.6). We thus changed each one of the two parameters, τ or T, to improve the fitness of the simple model. The simple model was well fitted when T was set to be 2.14 s (Fig 9B, bottom, thick line; $R^2 = 0.89$ where $\tau = 2$ s and $T = 2.14$ s), but the change of τ did not gave good fitting ($R^2 = -1.12$ where $\tau = 0.6$ s and $T = 0.3$ s [best fit]). The simple model could be applied to RP in the dorsomedial striatum only if the dead time T was redetermined, suggesting the subregion dependence of the dead time in the striatum.

He et al. also discovered cortical RP that has a 5-s time window for norepinephrine (NE) signal after pre–post pairing (Fig 9C) [3]. They also showed that NE activates G_s-coupled β2AR. G_s is similar to G_{olf}; thus, the RP should be based on G_s and Ca^{2+}-dependent AC activity, to which the simple model was applicable (Fig 9C). The simple model with the original parameters did not show a good fit (Fig 9C, bottom; dashed line; R^2, 0.68), but gave a good fit when T was set to be 7.5 s (Fig 9C, bottom; thick line; $R^2 = 0.93$ where $\tau = 2$ s and $T = 7.5$ s). The change of τ also improved the fitting ($R^2 = 0.77$ where $\tau = 5.06$ s and $T = 0.3$ s [best fit]). In both cases, the longer time lags gave the better fitting. Those results suggest the requirement of brain region-dependent rate constant of AC1 for the time window for RP.

References

1. Yagishita S, Hayashi-Takagi A, Ellis-Davies GCR, Urakubo H, Ishii S, Kasai H. A critical time window for dopamine actions on the structural plasticity of dendritic spines. Science. 2014; 345(6204): 1616-1620. pmid: ISI:000342164500047.
2. Shindou T, Shindou M, Watanabe S, Wickens J. A silent eligibility trace enables dopamine-dependent synaptic plasticity for reinforcement learning in the mouse striatum. Eur J Neurosci. 2019; 49(5): 726-736. pmid: 29603470.
3. He K, Huertas M, Hong SZ, Tie X, Hell JW, Shouval H, et al. Distinct Eligibility Traces for LTP and LTD in Cortical Synapses. Neuron. 2015; 88(3): 528-538. pmid: 26593091.
4. Brzosko Z, Schultz W, Paulsen O. Retroactive modulation of spike timing-dependent plasticity by dopamine. Elife. 2015; 4. pmid: 26516682.
5. Fisher SD, Robertson PB, Black MJ, Redgrave P, Sagar MA, Abraham WC, et al. Reinforcement determines the timing dependence of corticostriatal synaptic plasticity in vivo. Nat Commun. 2017; 8(1): 334. pmid: 28839128.
